# A Rare Case of* Streptococcus alactolyticus* Infective Endocarditis Complicated by Septic Emboli and Mycotic Left Middle Cerebral Artery Aneurysm

**DOI:** 10.1155/2016/9081352

**Published:** 2016-07-21

**Authors:** Patricia Almeida, Jaclyn Railsback, James Benjamin Gleason

**Affiliations:** ^1^Department of Internal Medicine, Cleveland Clinic Florida, Weston, FL 33331, USA; ^2^Department of Pulmonary and Critical Care Medicine, Cleveland Clinic Florida, Weston, FL 33331, USA

## Abstract

To date,* S. alactolyticus *endocarditis complicated by middle cerebral artery aneurysm has not been reported. We describe the case of a 65-year-old female with a history of hypertrophic cardiomyopathy with left ventricular outflow tract obstruction presenting with confusion and a apical holosystolic murmur. Angiography of the brain identified new bilobed left middle cerebral artery aneurysm. Serial blood cultures grew* S. alactolyticus*, and aortic and mitral valve vegetation were discovered on transesophageal echocardiography. The patient was treated with antimicrobial therapy, mitral and aortic valve replacements, and microsurgical clipping of cerebral aneurysm. This case serves to highlight the pathogenicity of a sparsely described bacterium belonging to the heterogenous* S. bovis* complex.

## 1. Introduction

*S. equinus/S. bovis* complex is a commensal group of nonenterococcal Group D* Streptococcus* species found within human and animal gastrointestinal tracts [1]. Certain species within the* S. equinus/S. bovis* complex, notably* S. gallolyticus *and* S. infantarius*, are known to cause human infections, such as bacteremia, meningitis, and endocarditis, and have a well-established association with colorectal carcinoma.* S. alactolyticus* is a rarely isolated species that constitutes the solitary species within DNA cluster IV of the* S. equinus/S. bovis* complex [2]. It has been isolated in the intestinal flora of pigs, chickens, pigeons, and canines, making it an almost exclusively animal-associated species [3–5]. Herein we describe the first case of* S. alactolyticus *endocarditis, complicated by septic emboli and mycotic cerebral aneurysm, in an immunocompetent human host.

## 2. Case Presentation

A 65-year-old Caucasian female presented to our institution with confusion and fevers. Her past medical history was notable for hypertension, hyperlipidemia, hypertrophic cardiomyopathy with left ventricular outflow tract obstruction, and mitral regurgitation. She had no prior surgeries but underwent routine dental cleaning four months prior to her presentation. Shortly after her dental procedure she developed episodic confusion and fevers prompting a brief admission to an outside medical facility where she underwent an unremarkable workup, including neurologic imaging, echocardiography, and blood cultures. She was ultimately discharged home with a ten-day course of levofloxacin to treat suspected bacteremia.

**Figure 1:**
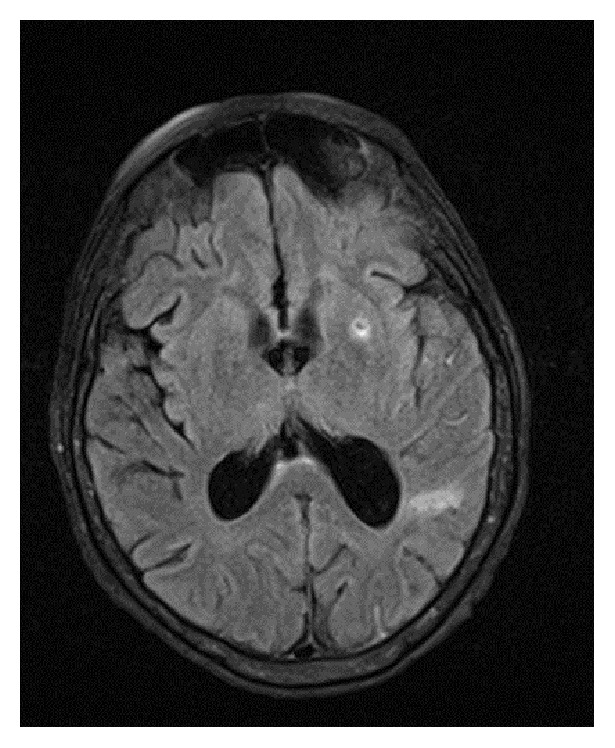
MRI of the brain demonstrating punctate areas of intraparenchymal hemorrhage.

**Figure 2:**
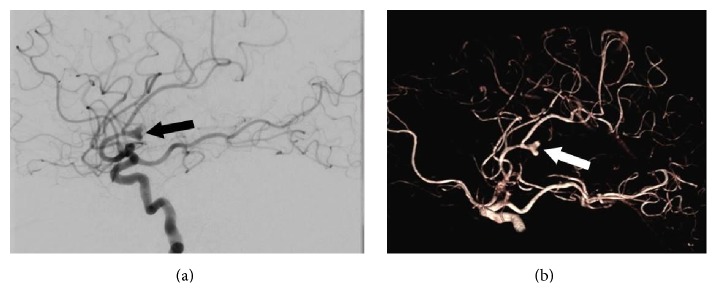
(a) Cerebral angiogram demonstrating left middle cerebral artery M2 segment mycotic aneurysm (black arrow). (b) 3D volume-rendering technique of the same aneurysm (white arrow).

Eight weeks after discharge her symptoms worsened and she presented to our facility. She was lethargic and disoriented. Initial vital signs were remarkable for normal blood pressure, respiratory rate, and oxygen saturation on room air. She had resting tachycardia of 106 beats per minute and was normothermic. Physical examination revealed a grade 3/6 holosystolic murmur, as well as an intention tremor of the bilateral upper extremities. Laboratory evaluation revealed a normocytic anemia and a positive rheumatoid factor. Computed tomography (CT) of brain revealed mild enlargement of lateral ventricles disproportionate to the degree of volume loss. Lumbar puncture was performed, of which cerebrospinal fluid bacterial cultures, cytology, and viral PCR studies were negative. Records from her previous hospitalization were obtained and expert review of her previous echocardiogram raised suspicion for native valve infective endocarditis of both the aortic and mitral valves. Empiric antimicrobial therapy with vancomycin, gentamicin, and penicillin G was initiated, with noticeable improvement in the patient's mentation within 48 hours. Serial blood cultures grew* Streptococcus alactolyticus. *Magnetic resonance imaging (MRI) of the brain revealed numerous areas of intraparenchymal and subarachnoid hemorrhage (Figure 1) concerning septic emboli. Cerebral angiogram showed left middle cerebral artery M2 segment mycotic aneurysm (Figure 2). Transesophageal echocardiogram (TEE) confirmed large mobile mitral and aortic valve vegetation with severe mitral and aortic regurgitation. CT of abdomen and pelvis showed new bilateral renal and splenic lesions, representing multiple small infarcts. Based on the* Streptococcus alactolyticus *sensitivities, antimicrobial therapy was deescalated to ceftriaxone, which was continued to complete a six-week course. She continued to have clinical improvement and her repeat blood cultures were negative. She was then treated with bioprosthetic aortic and mitral valve replacements. After an uncomplicated recovery from surgery she underwent definitive neurosurgical treatment of the left MCA mycotic aneurysm by left pterional craniotomy and microsurgical clipping. She recovered and was discharged to a rehabilitation facility with arrangements for outpatient colonoscopy to evaluate colonic malignancy.

## 3. Discussion

*S. equinus/S. bovis* complex, a group of nonenterococcal streptococci, is frequently found within the gastrointestinal tracts of humans and animals [1]. This complex has been isolated in 29–55% of individuals with Inflammatory Bowel Disease and colon cancer [6] and is responsible for up to 13% of cases of native valve endocarditis [7, 8]. However, it is worth noting that multiple genospecies exist within* S. equinus/S. bovis* complex and that the classically described association of* S. equinus/S. bovis* complex with colorectal cancer and native valve infective endocarditis does not seem to apply to all genospecies. These differences have caused some confusion in the classifications. Even so, performing a colonoscopy to evaluate colonic malignancy remains the prudent choice until more is known about the characteristics of the genospecies within this complex.

Efforts to streamline the nomenclature within the* S. equinus/S. bovis* complex have been undertaken, including PCR analysis of 16S ribosomal DNA sequences. These phylogenetic analyses have delineated the* S. equinus/S. Bovis *into four DNA homology clusters [2].* S. alactolyticus*, the solitary genospecies in DNA cluster IV, is the infectious pathogen isolated in the case described.

The more prevalent* S. gallolyticus*, comprising DNA cluster III of the* S. equinus/S. bovis *complex, is a well-known inhabitant of the human gut microbiota; hence, it is the species most notoriously associated with native valve infective endocarditis and colon cancer [2].* S. alactolyticus*, as opposed to* S. gallolyticus*, is almost an exclusively animal-associated species and has been isolated from the intestinal flora of pigs, chickens, pigeons, and canines [3–5]. Human reports of* S. alactolyticus* infection are exceedingly rare. Most recently, Toepfner et al. described a fatal case of fulminant neonatal sepsis and respiratory distress syndrome due to this pathogen [9].

Our patient presented with renal, splenic, and intracerebral infarcts, as well as an intracerebral mycotic aneurysm, in the setting of* S. alactolyticus* bacteremia and native valve left-sided endocarditis. Research suggests that septic emboli occur in 22–43% of patients with infective endocarditis [10] and can cause irreversible end-organ damage throughout the body by occlusion of the arterial lumen. Despite the high rates of embolism, only 1–5% of patients develop symptomatic mycotic aneurysms [11]. The most common locations for mycotic aneurysms include intracranial, visceral abdominal, and extremity arteries, with predisposition for involving sites of bifurcation [11]. Given their virulent nature* Staphylococcus* spp. and* Salmonella *spp. are the two most common causative pathogens. However, our case illustrates the first reported case of symptomatic left middle cerebral mycotic aneurysm caused by* S. alactolyticus*, a scarcely virulent species. One of the most catastrophic complications of mycotic aneurysms is subarachnoid hemorrhage and aneurysmal rupture, the latter accounting for 80% mortality rate [12]. Despite the stated risks, our patient underwent bioprosthetic aortic and mitral valve replacements requiring heparinization, followed by microsurgical clipping of the left MCA mycotic aneurysm.

## 4. Conclusion

Despite historical clinical practice, it has been demonstrated that there is significant heterogeneity in the clinical manifestations of members within the* S. equinus/S. bovis* complex. We present a unique case in which* S. alactolyticus*,* S. equinus/S. bovis* complex subspecies primarily associated with animal pathogenicity, caused life-threatening infection in an immunocompetent adult female. This group should no longer be perceived as a single bacterial entity, as routine efforts to identify specific* S. equinus/S. bovis* complex subspecies will aid in identification and recognition of their unique clinical characteristics.
